# Evaluating the Effect of Cerebrolysin as an Adjuvant to Standard Therapy in Patients with Acute Ischemic Stroke: A Prospective Observational Study

**DOI:** 10.3390/medicina61091531

**Published:** 2025-08-26

**Authors:** Geetha Kandasamy, Vijayakumar Arumugam, Khalid Orayj, Asma M. Alshahrani, Tahani S. Alanazi, Amjad Hmlan, Jithin P. Venugopal

**Affiliations:** 1 Department of Clinical Pharmacy, College of Pharmacy, King Khalid University, Abha 61421, Saudi Arabia; glakshmi@kku.edu.sa (G.K.); amhamln@kku.edu.sa (A.H.); 2 Head Pharmacy Services, Kovai Medical Center and Hospital, Coimbatore 641014, India; 3 Department of Clinical Pharmacy, College of Pharmacy, Shaqra University, Dawadimi 11961, Saudi Arabia; 4 Department of Pharmacy Practice, KMCH College of Pharmacy, Coimbatore 641048, India

**Keywords:** acute ischemic stroke, cerebrolysin, Barthel index, functional outcome, NIH stroke scale, adjuvant therapy, standard therapy

## Abstract

*Background and Objectives:* Acute ischemic stroke is a major cause of disability and mortality. Cerebrolysin, a neuropeptide with neuroprotective and neurotrophic properties, may enhance post-stroke recovery. This study evaluated the impact of adding Cerebrolysin to standard therapy on clinical outcomes in patients with acute ischemic stroke. *Materials and Methods:* This non-randomized prospective observational study included 143 adults with acute ischemic stroke at Kovai Medical Center and Hospital, Coimbatore (April 2016–May 2018). Participants were divided into two groups: the standard therapy group (*n* = 70) and the adjuvant therapy group *(n* = 73), which received Cerebrolysin (30 mL IV daily for 14 days) in addition to standard care. Stroke severity and functional outcomes were evaluated using the National Institutes of Health Stroke Scale (NIHSS) and Barthel Index (BI) at baseline and Day 14. A *p* < 0.05 was considered statistically significant. *Results:* Stroke severity improved in both groups, but the adjuvant group demonstrated significantly greater reductions in NIHSS scores from 9.90 ± 2.90 to 3.40 ± 1.40 compared to the standard group, which improved from 10.10 ± 2.80 to 4.80 ± 1.30 (*t* = 6.19, *p* < 0.001). Additionally, 43.84% of patients in the adjuvant group shifted to minor stroke severity versus 25.71% in the standard group. Both groups showed significant improvements across all domains of the BI, which assesses activities of daily living (ADL); however, the gains were consistently greater in the adjuvant group (*p* < 0.001). A higher proportion of patients in the Cerebrolysin group achieved slight dependency (38.36%) or full independence (16.44%), compared to 20% and 5.71% in the standard group, respectively. *Conclusions:* This prospective observational study suggests that adding Cerebrolysin to standard therapy was associated with greater neurological recovery and functional independence in acute ischemic stroke patients. However, the short follow-up, single-center setting, and lack of randomization limit generalizability. Larger multicenter randomized trials with longer follow-up are needed to confirm these findings.

## 1. Introduction

Stroke remains the second leading cause of death and the third leading cause of disability worldwide, according to the World Health Organization [1,2]. Ischemic stroke, which results from thrombotic or embolic occlusion of cerebral arteries, accounts for approximately 60–80% of all stroke cases [3]. The burden of stroke is disproportionately high in low- and middle-income countries, contributing to nearly 75% of global stroke-related deaths and around 81% of associated disabilities [4]. In India, stroke constitutes a significant public health concern, accounting for approximately 14% of the global disability-adjusted life years (DALYs) attributed to the disease [5]. The age-adjusted prevalence of stroke in India ranges from 84 to 262 per 100,000 in rural areas and from 334 to 424 per 100,000 in urban regions [6], with recent studies reporting an annual incidence of 119 to 145 per 100,000 individuals [7].

Given this growing burden, there is increasing interest in neuroprotective agents that may augment recovery following acute ischemic stroke. Cerebrolysin, a neuropeptide-based compound with both neurotrophic and neuroprotective properties, has shown promise in preclinical studies and early clinical trials [8,9]. Despite multiple randomized controlled trials demonstrating the efficacy of Cerebrolysin in acute ischemic stroke [10,11,12,13], several important gaps remain in the literature. Notably, high-quality real-world data assessing its effectiveness and safety as an adjuvant to standard therapy outside controlled trial settings are scarce, particularly in Indian populations where the stroke burden is substantial [13]. Furthermore, many existing studies are constrained by small sample sizes, methodological inconsistencies, and short follow-up durations. Critically, comparative data directly evaluating neurological and functional outcomes between standard therapy alone and combined therapy with Cerebrolysin in routine clinical practice are lacking [10]. This lack of robust real-world evidence restricts our understanding of Cerebrolysin’s practical benefits and hinders its integration into standardized stroke management protocols. Addressing these gaps through prospective observational studies is essential to inform clinical decision-making and improve patient outcomes.

Cerebrolysin has demonstrated promising results in the treatment of acute ischemic stroke across several recent clinical trials and meta-analyses. It has been shown to enhance neurological recovery and reduce impairments, particularly in patients with moderate to severe acute ischemic stroke [14]. Moreover, in patients who have undergone mechanical thrombectomy, Cerebrolysin as an adjuvant therapy significantly improves neurological and functional outcomes while reducing the rates of hemorrhagic transformation [15]. Nonetheless, most trials are conducted under controlled conditions that may limit generalizability to routine clinical practice.

Mechanistically, Cerebrolysin mimics endogenous neurotrophic factors, fostering neuroprotection and neuroplasticity following ischemic injury. Ischemic stroke induces a hostile cellular environment characterized by neuronal dysfunction and degeneration. Experimental models indicate that Cerebrolysin enhances neuronal sprouting and neurogenesis, facilitates neural network reconstruction, and preserves neuronal structures by inhibiting calpain activity. It also reduces infarct volume and promotes functional recovery [16,17,18]. These effects are mediated through key molecular pathways, including PI3K/AKT, GSK3β, and Sonic Hedgehog (Shh), which contribute to blood–brain barrier stability and neuroprotection [19,20,21].

Although clinical trials have reported favorable outcomes with Cerebrolysin, most were conducted under controlled conditions that may not reflect real-world clinical practice. For example, a multicenter, randomized, double-blind trial found that Cerebrolysin was well tolerated and significantly improved both neurological status and functional outcomes in the early recovery phase of acute ischemic stroke [12]. Supporting this, a pilot study of 44 patients with severe stroke National Institutes of Health Stroke Scale (NIHSS > 8) who failed reperfusion therapy reported better 12-month outcomes among those receiving Cerebrolysin, with 70% achieving a modified Rankin Scale (mRS) score of 0–3 compared to 48% in the control group (*p* = 0.1) [22]. Similarly, Poljakovic et al. observed that Cerebrolysin used as adjunctive therapy post-reperfusion was associated with lower mortality and reduced risk of symptomatic intracerebral hemorrhage [23].

Despite these promising findings, the generalizability of such results to routine clinical settings remains uncertain. The administration of post-stroke rehabilitation remains a complex challenge worldwide, as the degree of disability and the course of recovery following stroke are highly individualized and variable [24]. Consequently, there is a need for prospective observational studies to evaluate the real-world utility of Cerebrolysin across broader patient populations. The absence of such evidence limits its incorporation into standardized stroke care protocols.

Based on this background, we hypothesize that adding Cerebrolysin to standard therapy may facilitate post-stroke neuroplasticity and neuronal repair, thereby reducing stroke severity and enhancing functional recovery. While clinical trials have demonstrated promising results, there remains limited real-world evidence—particularly from Indian populations—directly comparing standard therapy alone with standard therapy plus Cerebrolysin in acute ischemic stroke. Therefore, this prospective observational study aimed to evaluate and compare the clinical outcomes of patients receiving standard therapy alone versus those receiving standard therapy combined with Cerebrolysin, focusing on neurological recovery and functional independence in a real-world Indian hospital setting.

## 2. Methods

### 2.1. Study Design and Setting

This non-randomized prospective open-label observational study was conducted from April 2016 to May 2018 at Kovai Medical Center and Hospital (KMCH), an 850-bed NABH-accredited tertiary care facility located in Coimbatore, Tamil Nadu. The study aimed to evaluate the effect of Cerebrolysin as an adjuvant to standard therapy on clinical outcomes in patients diagnosed with acute ischemic stroke.

### 2.2. Sample Size and Study Population

**Figure 1:**
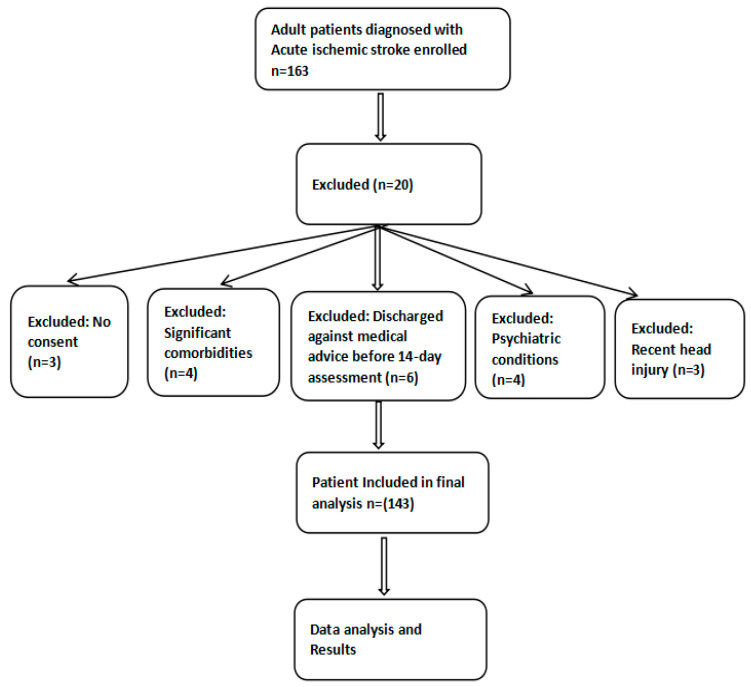
Flow chart illustrating participant screening, inclusion, and exclusion process.

An a priori power analysis was conducted using G*Power 3.1.9.7 to determine the minimum required sample size. Assuming a moderate effect size (Cohen’s d = 0.5), an alpha level of 0.05, and 80% power, a minimum of 64 participants per group was needed to detect significant differences in NIHSS scores and BI scores between groups. With 143 patients included (70 in the standard therapy group and 73 in the adjuvant therapy group), the study was adequately powered. A total of 163 adult patients diagnosed with acute ischemic stroke were initially enrolled, of whom 143 met the eligibility criteria and were included in the final analysis (Figure 1). This exploratory prospective cohort study included all eligible patients admitted during the defined study period. As this was a prospective observational study without randomization, treatment allocation was made based on the attending physician’s clinical judgment, taking into account patient or family preference, and aligned with standard hospital practice. Participants were subsequently categorized into two groups based on the treatment they received.

The standard therapy group (*n* = 70) received guideline-based pharmacological management for acute ischemic stroke, including thrombolytic agents (alteplase or tenecteplase—recombinant tissue plasminogen activators) for eligible patients within the therapeutic window, antiplatelet agents (aspirin and clopidogrel), lipid-lowering agents (statins—HMG-CoA reductase inhibitors), and anticoagulants when indicated (e.g., low molecular weight heparin or warfarin for cardioembolic stroke), along with supportive care including blood pressure control, glycemic management, and physiotherapy. The Adjuvant Therapy Group (*n* = 73) received the same standard therapy along with Cerebrolysin, administered as an intravenous infusion of 30 mL diluted in 100 mL of normal saline once daily for 14 consecutive days. Patients in the adjuvant therapy group received Cerebrolysin within 24 to 72 h of symptom onset. Concomitant medications were administered according to standard hospital protocols and included thrombolytic agents (alteplase, tenecteplase), osmotic agents (mannitol, hypertonic saline), antiplatelet agents (aspirin, clopidogrel), statins, anticoagulants, antihypertensives, and glucose-lowering agents as indicated. Both the standard therapy and adjuvant therapy groups received similar medications, and no intentional differences were introduced.

Eligible participants for this study were adults aged 18 years or older, of either gender, who were newly diagnosed with acute ischemic stroke confirmed by computed tomography (CT) or magnetic resonance imaging (MRI) and admitted within 24 h of symptom onset. Only those with a baseline NIHSS score between 4 and 20 were included, representing mild to moderate-to-severe stroke severity. Exclusion criteria included patients with hemorrhagic stroke and individuals with significant comorbidities such as chronic kidney disease, liver disease, myocardial infarction, congestive heart failure, or psychiatric disorders. Patients with severe renal or hepatic impairment, those discharged against medical advice, those with a history of epilepsy or recent head trauma, or those who were comatose at admission were also excluded. Patients with known hypersensitivity to Cerebrolysin or those who declined informed consent were excluded, as they were ineligible for adjuvant therapy.

### 2.3. Rehabilitation Protocol

All patients received routine inpatient rehabilitation services during hospitalization, including physical therapy, occupational therapy, and speech therapy, tailored to individual clinical needs. These services were accessible to both the standard therapy and adjuvant therapy groups. However, rehabilitation was not delivered through a standardized protocol and was administered at the discretion of the treating physician, based on the patient’s clinical condition. This individualized approach reflects real-world practice but may have introduced heterogeneity in the intensity and type of therapy provided.

### 2.4. Data Collection

Data were collected through structured case record forms, patient medical charts, and hospital records. The variables included demographic information such as age, gender, marital status, education level, body mass index (BMI), and history of smoking and alcohol use. Clinical data covered comorbid conditions including hypertension, diabetes mellitus, dyslipidemia, and ischemic stroke subtypes, along with laboratory investigations such as blood glucose levels, triglycerides, high-density lipoprotein (HDL), and low-density lipoprotein (LDL). Neurological and functional assessments were performed at baseline (Day 0) and on Day 14 post-treatment using NIHSS and the BI to evaluate stroke severity and functional independence, respectively. These assessments were conducted without blinding, as assessors were aware of participants’ treatment allocation, and no formal blinding procedures were implemented. Data on length of hospital stay (LOS), defined as the number of days from admission to discharge, were collected from hospital records for all participants. LOS was recorded to explore potential differences in hospitalization duration between the treatment groups.

### 2.5. Outcome Measures: The National Institutes of Health Stroke Scale (NIHSS)

NIHSS is a widely used clinical tool designed to assess the severity of neurological impairment in patients with acute stroke [25]. It includes 15 items, covering key neurological functions such as level of consciousness, visual fields, motor function, sensory deficits, coordination, language, and speech. Each item is scored on a scale, and the total NIHSS score ranges from 0 to 42, with higher scores indicating more severe neurological deficits. Stroke severity is categorized according to the following score ranges: a score of 0 indicates no symptoms, 1–4 denotes a minor stroke, 5–15 indicates a moderate stroke, 16–20 reflects a moderate to severe stroke, and 21–42 represents a severe stroke. The NIHSS was translated into the local language with the assistance of clinical and linguistic experts to ensure cultural and contextual relevance. The translated version was reviewed and refined for clarity and comprehension prior to implementation. Cronbach’s alpha was employed to assess internal consistency, yielding a value of 0.82 for the NIHSS, which indicates good reliability.

### 2.6. Barthel Index Scale (BI)

BI is a standardized and widely used instrument for assessing a patient’s functional independence in performing basic activities of daily living (ADLs), particularly in stroke and neurological rehabilitation [26]. It evaluates 10 key activities: feeding, bathing, grooming, dressing, bowel and bladder control, toilet use, transfers (e.g., from bed to chair), mobility on level surfaces, and stair climbing. Each activity is scored based on the level of assistance required: feeding (0, 5, or 10), bathing and grooming (0 or 5), dressing and toilet use (0, 5, or 10), bowel and bladder control (0, 5, or 10), transfers and mobility (0, 5, 10, or 15), and stair climbing (0, 5, or 10). A score of 0 indicates complete dependence, while higher scores reflect increasing independence. The total score ranges from 0 to 100, with interpretation as follows: 0–20 (total dependency), 21–60 (severe dependency), 61–90 (moderate dependency), 91–99 (slight dependency), and 100 (fully independent). The BI is valued for its simplicity, reliability, and sensitivity to functional changes over time. In this assessment, participants respond to ten questions about their physical capabilities, scoring each task as 0 (completely dependent), 5 (requires some assistance), or 10 (fully independent). The total BI score ranges from 0 to 100, with higher scores indicating greater functional independence and less disability. The BI was translated into the local language with input from both clinical and language experts to ensure cultural relevance and comprehension. Cronbach’s alpha was employed to assess internal consistency, yielding a value of 0.85 for the BI, which indicates good reliability.

### 2.7. Ethics Approval

The study protocol, including the off-label use of Cerebrolysin as an adjuvant therapy in acute ischemic stroke, was reviewed and approved by the Institutional Ethics Committee of KMCH (Registration No: EC/AP/459/03/2016), which is registered with the appropriate national regulatory authorities. The study was conducted in accordance with the ethical principles of the Declaration of Helsinki and its subsequent amendments. Written informed consent was obtained from all participants or their legally authorized representatives prior to enrollment. Confidentiality and anonymity were strictly maintained, and all data were used exclusively for research purposes.

### 2.8. Statistical Analysis

Data analysis was performed using IBM SPSS Statistics for Windows, Version 20.0 (IBM Corp., Armonk, NY, USA). Participant characteristics were summarized using descriptive statistics, with means and standard deviations (SD) reported for continuous variables and frequencies with percentages for categorical variables. For non-parametric data, medians and interquartile ranges (IQRs) were reported. Normality of continuous variables was assessed using the Shapiro–Wilk test. Between-group comparisons of laboratory parameters were performed using independent *t*-tests, while non-parametric data, including NIHSS and Barthel Index (BI) scores, were compared using the Mann–Whitney U test. Within-group comparisons (before and after therapy) were conducted using the Wilcoxon signed-rank test. Categorical variables were compared between groups using the chi-square test. For within-group changes in binary outcomes, such as a shift to independence, the McNemar test was applied. Bonferroni correction was applied for multiple comparisons to control the risk of type I error, and a *p*-value < 0.05 was considered statistically significant. Baseline differences between the Standard Therapy and adjuvant therapy groups were evaluated using standardized mean differences (SMDs) for key sociodemographic and clinical variables, providing a quantitative measure of group comparability, with smaller values indicating better balance. No formal propensity score matching was applied; however, SMDs were used to assess potential baseline imbalances.

## 3. Results

A total of 143 patients with acute ischemic stroke were enrolled in the study, comprising 70 in the standard therapy group and 73 in the adjuvant therapy group receiving Cerebrolysin. The baseline sociodemographic and clinical characteristics of both groups are presented in Table 1. Baseline characteristics were generally comparable between the standard therapy and adjuvant therapy groups, as reflected by the calculated SMDs. SMD values before and after adjustment indicated minimal differences across all sociodemographic and clinical variables, supporting the baseline comparability of the two groups. In terms of age distribution, the majority of patients in both groups were aged ≥ 65 years (60.0% in the standard group vs. 63.01% in the adjuvant group), followed by those aged 45–64 years. Only a small proportion of patients were under 45 years of age (8.57% vs. 2.74%). Male patients were predominant in both groups (68.57% in the standard group vs. 71.23% in the adjuvant group). Regarding educational status, over half of the patients in both groups had completed secondary school (52.86% vs. 53.42%), while approximately one-third had a university education (32.86% vs. 30.14%). Most patients were married, with only a few reporting being single or divorced (2.86% vs. 1.37%). Normality was assessed using the Shapiro–Wilk test (*n* = 143), which yielded a *W* value of 0.923 and a *p*-value of 0.67, indicating no significant deviation from normality. However, due to the ordinal nature of the NIHSS and BI scores, non-parametric tests were chosen for further analysis. In terms of comorbidities, the standard therapy group had a higher proportion of patients with ischemic stroke and hypertension alone (37.14%) compared to the adjuvant group (19.18%). Conversely, combined hypertension and diabetes was slightly more common in the adjuvant group (27.40% vs. 22.86%). A greater number of patients in the adjuvant group had no comorbidity or other conditions (38.36%) than in the standard group (22.86%). Analysis of social habits showed that a higher percentage of patients in the adjuvant group reported both alcohol and smoking use (38.36%) compared to the standard group (22.86%). Meanwhile, a larger proportion of patients in the standard group reported no such habits (42.86% vs. 36.99%). Regarding the types of ischemic stroke, small-vessel occlusion (lacunar stroke) was the most common subtype in both groups (42.86% in the standard group vs. 43.84% in the adjuvant group), followed by large artery atherosclerosis and cardioembolism. Other and undetermined etiologies were less frequently observed. The median length of hospital stay was similar between the two groups, with a median of 15 days (IQR 14–17) in the standard therapy group and 15 days (IQR 14–19) in the adjuvant therapy group. There were no statistically significant differences between the standard and adjuvant therapy groups in baseline characteristics, including age (*p* = 0.314), gender (*p* = 0.728), education (*p* = 0.907), and marital status (*p* = 0.535). Comorbidity profiles (*p* = 0.062), social habits (*p* = 0.072), and types of ischemic stroke (*p* = 0.937) or length of stay (*p* = 0.071). No mortality was observed in either group during the study period.

Table 2 compares laboratory parameters and vital signs between the standard therapy group (*n* = 70) and the Adjuvant Therapy group (*n* = 73). A significantly lower pulse rate was observed in the adjuvant group (79.40 ± 6.25) compared to the standard group (81.45 ± 5.56; *p* = 0.040). No significant differences were found in triglycerides (*p* = 0.105), HDL (*p* = 0.304), LDL (*p* = 0.213), systolic BP (*p* = 1.000), diastolic BP (*p* = 0.778), or random blood sugar (*p* = 0.408).

**Figure 2:**
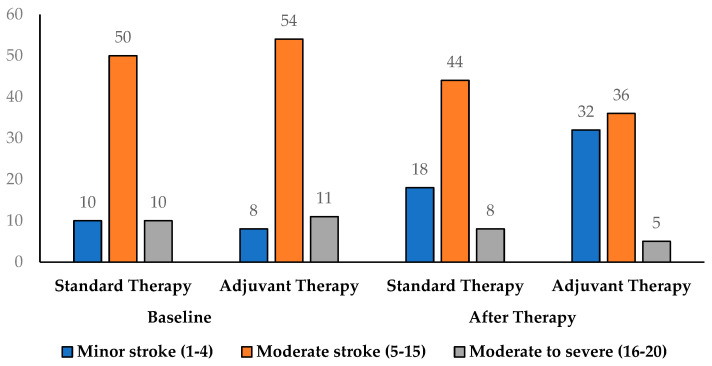
Stroke Severity Based on NIHSS Scores Before and After Treatment in the Study Population (*n* = 143).

Table 3 illustrates the distribution of stroke severity, classified using the NIHSS score, before and after treatment in both the Standard Therapy and Adjuvant Therapy (Cerebrolysin) groups. At baseline, the majority of patients in both groups presented with moderate stroke (NIHSS 5–15), accounting for 71.43% in the standard therapy group and 73.97% in the Adjuvant Therapy group. Minor stroke cases (NIHSS 1–5) were less common, comprising 14.29% and 10.96% of patients in the standard and adjuvant groups, respectively, while moderate to severe strokes (NIHSS 15–20) made up 14.29% and 15.07%, respectively. Following treatment, both groups showed improvement, with an increase in the proportion of patients classified as having minor strokes. In the standard therapy group, the proportion of minor stroke cases increased from 14.29% to 25.71%, while in the Adjuvant Therapy group, it increased markedly from 10.96% to 43.84%. Concurrently, the percentage of patients with moderate to severe strokes declined from 14.29% to 11.43% in the standard group and from 15.07% to 6.85% in the adjuvant group Figure 2. Between group comparison after treatment showed a trend toward better outcomes in the Adjuvant Therapy group, although the difference did not reach statistical significance (*p* = 0.069). Within-group analysis using McNemar’s test for binary shift toward independence demonstrated significant improvement in both groups (*p* = 0.0078 for Standard Therapy and *p* < 0.0001 for Adjuvant Therapy). While both therapies reduced stroke severity, the magnitude of improvement was greater in the Adjuvant Therapy group, indicating a more substantial shift toward minor stroke status and suggesting potential clinical benefit with the addition of Cerebrolysin.

Table 4 presents a comparison of stroke severity, as measured by the NIHSS scores, between the Standard Therapy and Adjuvant Therapy (Cerebrolysin) groups. At baseline, the median NIHSS score was significantly higher in the standard therapy group (11, IQR 9–12) compared with the Adjuvant Therapy group (5, IQR 4–6) (mean difference = 0.2; 95% CI −0.742 to 1.142; *p* = 0.032). Following treatment, both groups demonstrated significant reductions in NIHSS scores. The median NIHSS score decreased from 11 to 10 in the standard therapy group and from 5 to 3 in the Adjuvant Therapy group. The magnitude of improvement was significantly greater in the Adjuvant Therapy group (*p* = 0.046). These results indicate that Cerebrolysin, when used as an adjuvant to standard therapy, may lead to a greater reduction in stroke severity among patients with acute ischemic stroke. These findings suggest that Cerebrolysin, when used as an adjuvant to standard therapy, may lead to a more pronounced reduction in stroke severity among patients with acute ischemic stroke.

Table 5 presents the comparison of BI scores at baseline and after therapy among patients receiving standard therapy and those receiving adjuvant therapy with Cerebrolysin. Within-group analysis revealed significant improvement across all 10 functional domains in both groups (*p* < 0.001 for all). Between-group comparisons indicated that, after Bonferroni adjustment, the adjuvant therapy group achieved significantly greater improvements in dressing (median difference = –1; 95% CI: –2.0 to 0.0; adjusted *p* = 0.0299), bowel control (median difference = –1; 95% CI: –2.0 to 0.0; adjusted *p* = 0.0099), transfers (median difference = –1; 95% CI: –3.0 to 1.0; adjusted *p* = 0.0367), and stair climbing (median difference = –3; 95% CI: –4.0 to –1.5; adjusted *p* < 0.001). Differences in feeding, bathing, grooming, bladder control, toilet use, and mobility were not statistically significant after adjustment. These findings suggest that while both treatment strategies significantly improved functional performance, adjuvant Cerebrolysin conferred additional benefits in specific domains, particularly dressing, bowel control, transfers, and stair-climbing ability.

**Figure 3:**
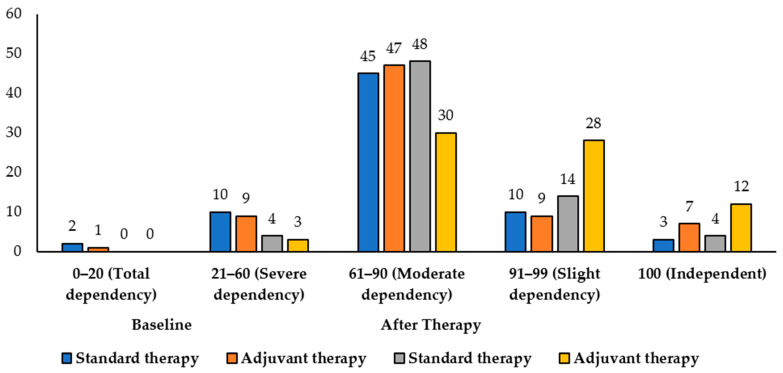
Functional outcome based on Barthel Index scores among standard and adjuvant therapy groups (*n* = 143).

Table 6 shows how patients in the standard therapy and adjuvant therapy (Cerebrolysin) groups scored on the BI, which measures their functional outcomes. At baseline, the majority of patients in both groups exhibited moderate dependency (61–90), with 45 (64.29%) in the standard therapy group and 47 (64.38%) in the adjuvant therapy group. Total dependency (0–20) was rare, observed in 2 (2.86%) patients receiving standard therapy and 1 (1.37%) in the adjuvant therapy group. Severe dependency (21–60) was present in 10 (14.29%) and 9 (12.33%) patients in the standard and adjuvant groups, respectively, while slight dependency (91–99) was seen in 10 (14.28%) and 9 (12.32%) patients. A few patients were fully independent (100), with 3 (4.28%) in the standard therapy group and 7 (9.58%) in the adjuvant therapy group. There was no significant difference between the groups at baseline (chi-square *p* = 0.732). After therapy, functional outcomes improved in both groups. In the standard therapy group, patients with moderate dependency increased slightly to 48 (68.57%), slight dependency increased to 14 (20%), and fully independent patients were 4 (5.71%). Severe and total dependency decreased to 4 (5.71%) and 0, respectively. In the adjuvant therapy group, a larger improvement was observed: moderate dependency decreased to 30 (41.10%), slight dependency increased to 28 (38.36%), and fully independent patients increased to 12 (16.44%). Severe and total dependency were reduced to 3 (4.11%) and 0, respectively, Figure 3. The After therapy distribution between the groups showed a statistically significant difference (chi-square *p* = 0.0048). Analysis of binary shift to independence using the McNemar test revealed no significant change in the standard therapy group (*p* = 0.642), whereas the adjuvant therapy group showed a significant improvement in independence after therapy (*p* = 0.04113).

## 4. Discussion

This prospective observational study evaluated the clinical effectiveness of Cerebrolysin as an adjuvant to standard therapy and conventional rehabilitation in patients with acute ischemic stroke. Our findings indicate that the addition of Cerebrolysin resulted in significantly greater improvements in both neurological and functional outcomes compared to standard therapy alone. Our results are consistent with a 2018 meta-analysis of nine randomized clinical trials, which demonstrated that Cerebrolysin administered in doses of 30 to 50 mL over 10–21 days significantly outperformed placebo, regardless of whether treatment was initiated immediately or with a seven-day delay [11,12,22,27,28,29]. While prior research has focused primarily on the acute phase, our study adds to the evidence supporting its efficacy in the early recovery phase of stroke rehabilitation [30,31]. The practical benefit of Cerebrolysin is further illustrated by one of our cases in which the patient regained independence in daily activities and ambulation. These improvements reflect those reported in a previous case study [32] involving a 10-day course of 30 mL/day, the minimum effective dose, which produced similar functional gains. This is consistent with the Copenhagen Stroke Study [33], which found that 95% of patients achieved functional recovery within 12.5 weeks of stroke onset.

According to Adams et al. [34] and related studies, individuals with a baseline NIHSS score of 15 have only a 23% chance of achieving full recovery three months post-stroke. The neuroprotective and neurotrophic properties of Cerebrolysin, which mimic endogenous neurotrophic factors [35,36], are believed to underlie its clinical efficacy. In our cohort, patients receiving Cerebrolysin demonstrated significantly greater reductions in NIHSS scores and higher improvements in BI scores, indicating earlier recovery and superior neurological and functional outcomes compared to standard therapy alone. Consistent with previous studies, Cerebrolysin has shown beneficial effects in acute ischemic stroke, supported by its neuroprotective actions, good safety and tolerability profile, and potential synergistic benefits when combined with thrombolytic therapy [12,22,37,38,39,40].

A comprehensive meta-analysis confirms that Cerebrolysin has a favorable safety profile in patients treated after acute ischemic stroke compared to placebo [41]. Additionally, a study investigating Cerebrolysin combined with early neurorehabilitation demonstrated significant improvements in overall neurological status and disability, with outcomes that can be readily applied in current clinical practice [42]. Furthermore, another meta-analysis supports previous findings that Cerebrolysin effectively alleviates early global neurological deficits in patients with acute ischemic stroke [27]. Additional evidence comes from major trials: the CASTA trial [11] reported a three-point greater improvement in NIHSS scores at day 90 among patients with baseline scores > 12 treated with Cerebrolysin versus placebo; the ECOMPASS study [43] found better motor recovery in patients with severe deficits (Fugl–Meyer Assessment < 50); and another study [44] observed significant BI score improvements, with 63.33% of patients progressing from severe to moderate dependency, reflecting regained physical function and increased independence. A retrospective study [30] involving 50 patients also showed that a 30-day course of 10 mL intramuscular Cerebrolysin, alongside standard rehabilitation, significantly reduced spasticity and improved strength and function compared to control. The 2021 guidelines from the European Academy of Neurology (EAN) and the European Federation of Neurorehabilitation Societies (EFNR), which recommend Cerebrolysin (30 mL/day intravenously for at least 10 days) as a pharmacological adjunct to early motor rehabilitation post-stroke [45]. These recommendations were developed by a multinational task force using the GRADE framework. Additionally, the 2020 updated guideline from the German Society of Neurorehabilitation recommends Cerebrolysin for 21 days, preferably within 24 to 72 h post-stroke, to support motor recovery, particularly for upper limb function [46].

However, our findings should be interpreted in the context of existing high-level evidence. A major Cochrane review by Ziganshina et al. [40] concluded that current randomized trial data do not support the routine use of Cerebrolysin in acute ischemic stroke, showing little or no effect on all-cause mortality or serious adverse events, but a possible increase in non-fatal serious adverse events. The authors called for well-designed, adequately powered trials to clarify efficacy and safety. In contrast, our real-world study found significant neurological and functional improvements with Cerebrolysin as an adjunct to standard therapy, without major safety concerns. These differences may reflect variations in patient populations, treatment timing, dosing, and concurrent rehabilitation. The Cochrane findings highlight the need for cautious interpretation of observational results and for confirmatory randomized trials in diverse, high-burden settings such as India.

Although baseline comorbidity rates differed slightly between the groups—particularly in the ‘No comorbidity/Other’ category (38.36% in the adjuvant group vs. 22.86% in the standard group)—SMDs for this variable indicated minimal imbalance. Nevertheless, this difference may introduce some selection bias, and caution should be exercised when interpreting the results. Comorbidities, particularly diabetes, remain clinically important, as they independently influence functional recovery and the risk of intercurrent illnesses during stroke rehabilitation [47,48]. Future studies using randomized designs or formal propensity score matching could further minimize potential bias and strengthen the robustness of findings.

Although income and occupation were not collected, educational status was used as a proxy for socioeconomic background. Educational level, which affects health literacy and rehabilitation engagement, was balanced between groups, suggesting minimal impact on outcomes [49,50]. Future studies could include broader socioeconomic measures to better assess their influence on stroke recovery. Although Cerebrolysin is officially approved in Austria and supported by European guidelines, its use in India, where this study was conducted, reflects growing clinical interest and off-label adoption supported by emerging evidence. This study supports the use of Cerebrolysin as an effective adjunct in early stroke rehabilitation. Significant improvements in NIHSS scores and BI scores and a higher proportion of patients achieving minimal dependency suggest that Cerebrolysin can accelerate recovery, improve functional independence, and potentially reduce long-term disability. Further randomized controlled trials are needed to evaluate the efficacy and safety of Cerebrolysin across all stages of stroke recovery, especially during the late subacute to chronic phases. Growing evidence from clinical trials suggests that Cerebrolysin treatment may offer significant benefits to the broader patient population.

### Limitations

This study has several important limitations. First, the non-randomized and observational design, with treatment allocation based on physician judgment and patient or family preference, limits the ability to draw causal inferences and introduces the risk of selection bias and residual confounding, despite the groups being generally comparable based on standardized mean differences (SMDs). Second, the study was conducted at a single tertiary care center with a relatively modest sample size (*n* = 143), potentially limiting the generalizability of the findings to other populations or healthcare settings. The strict exclusion criteria, particularly the omission of patients with common comorbidities, further reduce the applicability of the results to real-world clinical practice. Third, the study exclusively focused on patients with acute ischemic stroke, deliberately excluding those with hemorrhagic stroke due to their distinct pathophysiology and treatment responses. While this enhances internal validity, it restricts the generalizability of the findings to the broader stroke population. Educational status was used as a proxy for socioeconomic background, but detailed income and occupation data were not collected. Socioeconomic factors may influence health literacy, access to care, and rehabilitation engagement, potentially affecting outcomes. Future studies should include broader socioeconomic measures to better assess their impact on stroke recovery. A key limitation is the short follow-up, with outcomes assessed only up to 14 days during the acute inpatient phase, as most participants were discharged by or before this time. This restricts the ability to evaluate medium- or long-term recovery, and future studies with extended follow-up are needed to determine whether these early benefits are sustained. Additionally, variations in rehabilitation practices, including physical, occupational, and speech therapy, were not standardized and were provided at the discretion of the treating physician, which may have introduced variability that could influence functional recovery outcomes. Standardizing rehabilitation protocols in future studies could reduce such heterogeneity. Furthermore, Cerebrolysin is not approved by the European Medicines Agency (EMA) for the treatment of stroke, and current evidence from high-quality randomized controlled trials remains limited. This should be considered when interpreting the findings, and further robust, multicenter trials are warranted to confirm these results. Finally, outcome assessments using the NIHSS and BI were conducted without blinding, as assessors were aware of participants’ treatment allocation. No formal blinding procedures were implemented, which may have introduced measurement bias, particularly for outcomes involving subjective clinical judgment. Finally, no formal propensity score matching or multivariate adjustment was performed, which could have further minimized potential confounding. Future randomized studies or those employing robust confounding control techniques are warranted to confirm these results.

## 5. Conclusions

This prospective observational study suggests that adding Cerebrolysin to standard therapy was associated with improved neurological recovery and greater functional independence in acute ischemic stroke patients. These findings suggest that Cerebrolysin may enhance both neurological recovery and functional independence. While these findings are promising, they should be interpreted with caution due to the study’s non-randomized design, short follow-up, and single-center setting. Large multicenter randomized controlled trials with longer follow-up are needed to confirm causality, long-term effectiveness, and generalizability.

## Figures and Tables

**Table 1:** Sociodemographic and Clinical Characteristics of Patients Receiving Standard Therapy Versus Adjuvant Therapy with Cerebrolysin.

Variables	Category	Standard Therapy *n* (%) (70)	Adjuvant Therapy *n* (%) (73)	SMD Before	SMD After	*p* Value
**Age**	<45 years	6 (8.57)	2 (2.74)	−0.025	0.274	0.314
	45 to 64 years	22 (31.43)	25 (34.25)			
	≥65 years	42 (60)	46 (63.01)			
**Gender**	Male	48 (68.57)	52 (71.23)	0.124	−0.009	0.728
	Female	22 (31.43)	21 (28.77)			
**Education**	Primary school	10 (14.29)	12 (16.44)	−0.227	0.004	0.907
	Secondary school	37 (52.86)	39 (53.42)			
	University	23 (32.86)	22 (30.14)			
**Marital Status**	Married	68 (97.14)	72 (98.63)	−0.065	0.069	0.535
	Single/divorced	2 (2.86)	1 (1.37)			
**Comorbidity**	Ischemic stroke with hypertension	26 (37.14)	14 (19.18)	0.119	−0.006	0.062
	Ischemic stroke with diabetes	12 (17.14)	11 (15.07)			
	Ischemic stroke with hypertension and diabetes	16 (22.86)	20 (27.40)			
	No comorbidity/Other	16 (22.86)	28 (38.36)			
**Social habits**	Alcohol	8 (11.43)	2 (2.74)	−0.019	0.007	0.072
	Smoking	16 (22.86)	16 (21.92)			
	Alcohol and Smoking	16 (22.86)	28 (38.36)			
	None	30 (42.86)	27 (36.99)			
**Types of Ischemic strokes**	Large artery atherosclerosis (LAA)	18 (25.71)	20 (27.40)	−0.254	0.008	0.937
	Cardioembolism (CE)	12 (17.14)	14 (19.18)			
	Small-vessel occlusion (Lacunar)	30 (42.86)	32 (43.84)			
	Other determined etiology	4 (5.71)	3 (4.11)			
	Undetermined etiology	6 (8.57)	4 (5.48)			
**Length of stay (days)**	Length of stay (median (IQR))	15 (14–17)	15 (14–19)	0.212	0.045	0.071

SMD: Standardized mean differences, IQR: Interquartile range.

**Table 2:** Comparison of Laboratory Parameters and Vital Signs Between Standard Therapy and Adjuvant Therapy Groups.

Parameters	Standard Therapy (Mean ± SD) (*n* = 70)	Adjuvant Therapy (Mean ± SD) (*n* = 73)	t Value	*p* Value
Pulse (Beats/sec)	81.45 ± 5.56	79.40 ± 6.25	2.07	0.040 *
Triglyceride (mg/dl)	136.85 ± 47.73	125.14 ± 38.07	1.63	0.105
HDL (mg/dl)	45.25 ± 9.37	46.85 ± 9.62	−1.03	0.304
LDL (mg/dl)	109.97 ± 23.44	105.05 ± 20.02	1.25	0.213
Systolic BP (mmHg)	129.71 ± 6.63	129.71 ± 4.81	0.00	1.000
Diastolic BP (mmHg)	80.85 ± 7.01	80.57 ± 4.81	0.28	0.778
Random Blood Sugar level (mg/dl)	164.3 ± 42.5	158.7 ± 39.8	0.83	0.408

SD: Standard deviation, * Significant at 5% level.

**Table 3:** Stroke Severity Based on NIHSS Scores Before and After Treatment in the Study Population (*n* = 143).

Stroke Severity	Baseline		After Therapy	
	Standard Therapy *n* (%) (*n* = 70)	Adjuvant Therapy *n* (%) (*n* = 73)	Standard Therapy *n* (%) (*n* = 70)	Adjuvant herapy *n* (%) (*n* = 73)
Minor stroke (1–4)	10 (14.29)	8 (10.96)	18 (25.71)	32 (43.84)
Moderate stroke (5–15)	50 (71.43)	54 (73.97)	44 (62.86)	36 (49.32)
Moderate to severe (16–20)	10 (14.29)	11 (15.07)	8 (11.43)	5 (6.85)
*p* value (Chi squared)	0.835		0.069	

McNemar test for binary shift to independence: Standard *p* = 0.0078, Adjuvant *p* < 0.0001. NIHSS: National Institutes of Health Stroke Scale.

**Table 4:** Comparison of stroke severity by NIHSS scores among the study population (*n* = 143).

NIHSS Scores	Standard Therapy (70)	Adjuvant Therapy (73)	Mean Difference (Std—Adj)	95% CI for Mean Difference	Within-Group *p*-Value (Wilcoxon Signed-Rank Test)
Baseline	11 (9–12)	6 (5–7)	0.2	(−0.742, 1.142)	0.032 *
After therapy	10 (8–12)	3 (2–4)	1.4	(0.954, 1.846)	<0.0001 *

Between-group change *p*-value (Mann–Whitney U test): 0.046412 *. CI: Confidence interval; Std: Standard therapy, Adj- Adjuvant therapy * *p* < 0.05. NIHSS: National Institutes of Health Stroke Scale.

**Table 5:** Comparison of Individual Barthel Index Item Scores at Baseline and After Therapy Within and Between Standard and Adjuvant Therapy Groups with Bonferroni-Adjusted *p*-values (*n* = 143).

Item	Standard Therapy		Adjuvant Therapy		Within Group *p* Value (Standard Therapy)	Within Group *p* Value (Adjuvant Therapy)	Between group *p* Value	Median Difference in Change	95% CI for Median Difference in Change	Bonferroni Adjusted *p* Value
	Baseline Median (IQR)	After Therapy Median (IQR)	Baseline Median (IQR)	After Therapy Median (IQR)						
**Feeding**	4 (3–5)	9 (8–10)	5 (3–5)	9 (9–10)	<0.001	<0.001	0.201462	0	(−1.0, 1.0)	1.0
**Bathing**	2 (1–3)	4 (3–5)	2 (1–3)	5 (4–5)	<0.001	<0.001	0.226487	0	(−1.0, 1.0)	1.0
**Dressing**	4 (2–6)	9 (7–10)	4 (3–5)	9 (8–10)	<0.001	<0.001	0.012678	−1	(−2.0, 0.0)	0.029874533
**Grooming**	2 (2–3)	5 (4–5)	2 (2–3)	5 (4–5)	<0.001	<0.001	0.121933	0	(−1.0, 0.0)	1.0
**Bladder**	5 (3–6)	8 (7–10)	5 (4–7)	9 (8–10)	<0.001	<0.001	0.887038	0	(−1.0, 1.0)	1.0
**Bowels**	6 (5–8)	9 (8–10)	5 (4–7)	9 (8–10)	<0.001	<0.001	0.008754	−1	(−2.0, 0.0)	0.009875655908
**Toilet**	3 (2–6)	8 (7–10)	4 (3–6)	9 (8–10)	<0.001	<0.001	0.833918	0	(−1.0, 1.0)	1.0
**Mobility**	6 (3–8)	10 (8–13)	7 (4–10)	13 (11–14)	<0.001	<0.001	0.035672	−1	(−3.0, 1.0)	0.09853425456
**Transfers**	7 (5–9)	12 (10–13)	7 (5–10)	14 (12–15)	<0.001	<0.001	0.002356	−1	(−3.0, 1.0)	0.036744189012
**Stairs**	4 (2–6)	7 (5–9)	3 (1–5)	9 (8–10)	<0.001	<0.001	1.3 × 10^−5^	−3	(−4.0, −1.5)	0.0001330518418862441

Bladder: Bladder control, Bowels: Bowel control, and Stairs: Stair climbing. IQR: Interquartile range.

**Table 6:** Functional outcome based on Barthel Index scores among standard and adjuvant therapy groups (*n* = 143).

Score Range	Level of Dependency	Baseline		After Therapy	
		Standard Therapy (70) *n* (%)	Adjuvant Therapy (73) *n* (%)	Standard Therapy (70) *n* (%)	Adjuvant Therapy (73) *n* (%)
0–20	Total dependency	2 (2.86)	1 (1.37)	0	0
21–60	Severe dependency	10 (14.29)	9 (12.33)	4 (5.71)	3 (4.11)
61–90	Moderate dependency	45 (64.29)	47 (64.38)	48 (68.57)	30 (41.10)
91–99	Slight dependency	10 (14.28)	9 (12.32)	14 (20)	28 (38.36)
100	Independent	3 (4.28)	7 (9.58)	4 (5.71)	12 (16.44)
*p* value (Chi squared)		0.732		0.0048 *	

McNemar test for binary shift to independence: Standard *p* = 0.642, Adjuvant *p* = 0.04113 *.

## Data Availability

The original contributions presented in the study are included in the article, further inquiries can be directed to the corresponding author.
